# Wearables in Schizophrenia: Update on Current and Future Clinical Applications

**DOI:** 10.2196/35600

**Published:** 2022-04-07

**Authors:** Lakshan N Fonseka, Benjamin K P Woo

**Affiliations:** 1 Olive View-University of California Los Angeles Medical Center Sylmar, CA United States

**Keywords:** wearables, smartwatch, schizophrenia, digital phenotype, wearable, mHealth, mobile health, review, clinical application, clinical utility, clinical use, literature search, diagnosis, prevention

## Abstract

Schizophrenia affects 1% of the world population and is associated with a reduction in life expectancy of 20 years. The increasing prevalence of both consumer technology and clinical-grade wearable technology offers new metrics to guide clinical decision-making remotely and in real time. Herein, recent literature is reviewed to determine the potential utility of wearables in schizophrenia, including their utility in diagnosis, first-episode psychosis, and relapse prevention and their acceptability to patients. Several studies have further confirmed the validity of various devices in their ability to track sleep—an especially useful metric in schizophrenia, as sleep disturbances may be predictive of disease onset or the acute worsening of psychotic symptoms. Through machine learning, wearable-obtained heart rate and motor activity were used to differentiate between controls and patients with schizophrenia. Wearables can capture the autonomic dysregulation that has been detected when patients are actively experiencing paranoia, hallucinations, or delusions. Multiple platforms are currently being researched, such as Health Outcomes Through Positive Engagement and Self-Empowerment, Mobile Therapeutic Attention for Treatment-Resistant Schizophrenia, and Sleepsight, that may ultimately link patient data to clinicians. The future is bright for wearables in schizophrenia, as the recent literature exemplifies their potential to offer real-time insights to guide diagnosis and management.

## Introduction

Schizophrenia affects 1% of the world population and is associated with a reduction in life expectancy of 20 years [1]. The increasing prevalence of both consumer technology and clinical-grade wearable technology offers new metrics to guide clinical decision-making remotely and in real time [2-4]. These include standard measures, such as step count and sleep duration, but can expand through smartphone integration to include social activity, ambient light and noise sensing for sleep environment analysis, and others that will be discussed in this paper. Conversely, an increased amount of metrics can also be considered a disadvantage due to the challenge of translating the massive quantity of data generated from wearable devices into clinically relevant information. The solution for this may be using machine learning algorithms that are trained to identify pattern signatures and associate them with various clinical states, such as the onset of a manic or depressive episode.

The aim of this study is to review the recent literature on wearables and their potential clinical utility in schizophrenia, including their utility in first-episode psychosis (FEP) and diagnosis, relapse prevention, and patient safety. A literature search on PubMed was conducted for “((wearable) OR (smartwatch)) AND ((psychosis) OR (schizophrenia))” from 2018 to present. This time frame was selected because it allowed this paper to bridge the gap between prior systematic reviews and the most recent literature. Of the resulting 38 articles, 16 were either out of the scope of or not relevant to this paper. Thus, 22 of the 38 articles are presented in the context of the prior research they build upon, followed by a discussion on the future directions of this promising field.

## Ethical Considerations

In consideration of the vulnerable individuals with schizophrenia and the profoundly personal data being obtained, it is necessary to prioritize ethical principles in all related research [5,6]. Chivilgina et al [1] highlight the most urgent concerns, such as data confidentiality, the lack of clear safety standards, and insufficient evidence on the impact of wearable technology on self-perception. Establishing ethical guidelines for digital technology use among psychiatric patients, particularly for the continuous, unobtrusive, passive data collection performed by wearables, is a basic prerequisite for patient protection.

Recent studies have evaluated the perceptions of the psychiatric patient population toward wearable devices. Dewa et al [7] found that young people with a psychiatric history had a positive perception of wearables and the expectation that continuous detection should result in immediate responses. Specifically in schizophrenia, wearables were found to be acceptable by patients and did not induce any significant paranoia [8].

## Validity

Before wearables can have a significant clinical role, their measurements must first be validated. One obstacle is the wide variation in the devices used by researchers, as some studies use consumer products, such as the Fitbit (Fitbit LLC), while others opt for more standardized actigraphy measures that can offer increased accuracy at the cost of user convenience. Validating these methods specifically for sleep has been a recent focus in the literature, and the results are encouraging for most devices.

Several studies aimed to validate the sleep metrics of consumer devices, as wearables would be able to track sleep with minimal disturbance relative to polysomnography. Rookham et al [9] compared the Apple Watch (Apple Inc) to the clinically validated Philips Actiwatch Spectrum Pro (Koninklijke Philips NV) in 14 healthy adults. The study determined that the Apple Watch has an accuracy of 97% and sensitivity of 99% in identifying sleep, as well as a 79% specificity in detecting wakefulness. The watch tended to underestimate wakefulness after sleep onset by 5.74 minutes and overestimate total sleep time by 6.31 minutes. These results suggest that the Apple Watch is similar, in terms of sleep tracking capability, to the Philips actigraphy device, though future studies should include comparisons with the gold standard—polysomnography.

A 2021 article by Stucky et al [10] sought to validate the Fitbit Charge 2 (Fitbit LLC) against at-home polysomnography in 59 shift workers (police officers and paramedics). The Fitbit was found to overestimate rapid eye movement sleep latency by 29.4 minutes and wakefulness after sleep onset by 37.1 minutes. In addition, the Fitbit heart rate monitor showed limitations in detecting sudden heart rate changes due to decreased time resolution (ie, a decreased rate of gathering sleep data) when compared to polysomnography. The distribution of sleep episode durations was also different from the polysomnography results, and there were inaccuracies in sleep staging. These errors may have been due to the Fitbit proprietary algorithm, and they could be alleviated by having access to raw data that can be processed through open-source algorithms. Nonetheless, the study shows that the Fitbit can obtain reasonably accurate estimates of sleep and heart rate data.

These studies support prior literature that first demonstrated a lack of reliability with wearables, especially in the overestimation or underestimation of total sleep time and total wake time [11]. However, it was thought that these reliability issues may be partially due to the power of the studies, as they typically had less than 20 participants. Although the Stucky et al [10] study had 59 participants, sample sizes were otherwise consistently small across newer articles as well. Standardization and platforms for sharing data among researchers may have a role in solving this issue, and these will be detailed in further sections.

## Wearables in Schizophrenia Care

### Diagnosis and FEP

There is a bias in the literature toward feasibility studies and symptom monitoring rather than diagnosis or FEP identification [11,12]. This is likely because the process of psychiatric diagnosis typically involves an extended patient history, medication review, laboratory tests, and a period of behavioral observation [13]. However, although the use of wearables cannot replace the diagnostic process, real-time data have already proven to be promising in identifying physiological changes from baseline. Cella et al [14] studied 15 participants with FEP who wore a wrist wearable that recorded heart rate variability and electrodermal activity as proxies for distress. Participants also completed symptom self-assessments through a mobile phone app. The results showed that when distressing hallucinations and delusions were reported, electrodermal activity also significantly increased. Similarly, Schlier et al [15] found that when patients with schizophrenia were actively experiencing paranoia, alterations in the autonomic stress responses detected by wearables persisted until the paranoia subsided. No effects were found when patients reported hallucinations or intrusive thoughts. These results illustrate that the real-time detection of autonomic dysregulation may result in the earlier detection of psychiatric distress, which in turn may lead to having the clinical workup required for a diagnosis [15,16].

Additional advances are evident in a study by Reinertsen et al [17], in which changes in heart rate and locomotor activity were measured through a wearable patch in 16 patients with schizophrenia and 19 healthy controls. These patches measured signal complexity and interactions over time before the data were ultimately processed through a machine learning algorithm that allowed for perfect discrimination between controls and patients with schizophrenia.

Chen et al [18] used a novel privacy-preserving approach to using ambient noise levels as a measure of sociability. Wrist-worn audio bands recorded ambient noise over 1 week and classified signals based on the detected number of simultaneous speakers—a proxy for sociability. No speech content was analyzed to further protect privacy. Of 32 people, there were 8 outpatients with schizophrenia or schizoaffective disorders, 11 outpatients with major depressive disorder without psychotic features, and 13 controls. The resulting social ambience measure (SAM) allowed healthy controls to be distinguished from individuals with depressive or psychotic disorders, who spent more time in isolation with corresponding lower levels of social ambience. Participants had also completed the Patient Health Questionnaire-9 and Generalized Anxiety Disorder-7 forms prior to study enrollment, and the severity of depressive and anxiety symptoms corresponded with the SAM as well. Although the sample size and duration of the study were small, the feasibility of the SAM as an objective measure of sociability was clear. Immediate identification allows for timely interventions and dynamic reassessments, and the SAM may potentially detect behavioral precursors of FEP or depressive episodes.

Wearable actigraphy data processed through a machine learning algorithm using slope entropy was shown to be capable of significantly differentiating among depression, mania, and remission in patients with bipolar disorder [19]. The concept of slope entropy is significantly outside the scope of this article, but the ability to use wearable data to distinguish these states holds promise for the identification of prodromal patterns in the future. With 1 in 5 people owning a smartwatch in the United States, the identification of significant changes from baseline, such as insomnia or autonomic dysregulation, may be performed unobtrusively [20]. Some studies suggest that patients with schizophrenia tend to report sleep abnormalities prior to disease onset, and wearable data may one day alert clinicians of patients who warrant further psychiatric investigation [21].

### Management

Wearable data have the potential to be useful in guiding psychiatric management, particularly in the detection of relapse, which 80% of patients with schizophrenia are likely to encounter at least once within 5 years of FEP [22]. Lahti et al [23] provided 40 people with schizophrenia a wearable device to track activity levels and sleep and correlated these data with the severity of clinical symptoms, which was measured by scoring systems, such as the Positive and Negative Syndrome Scale (PANSS). The study was limited in terms of its results on relapse predictive value due to only 1 patient experiencing relapse. The authors stated that the relapse rate in the study was lower than a clinic’s relapse rate and was maybe affected by a selection bias, as less stable patients may have refused to participate in the study. Nevertheless, the study demonstrates the feasibility and acceptability of using wearables to monitor patients with schizophrenia.

Currently underway is a similar study of 100 individuals with schizophrenia spectrum disorders who were each given a Fitbit Charge 3 (Fitbit LLC) and smartphone for free to track multiple metrics over 1 year. These metrics include sleep, physical activity, ambient light, and finger taps [24]. This appears to be the first use of a finger tap metric in schizophrenia, though typing on a keyboard has been studied previously as a proxy for cognition in patients with bipolar disorder. Decreased accuracy in finger taps may represent impaired concentration during a depressed state [25]. The finger tap metric pairs well with the small screens of wearables and is an interesting measure for assessing cognitive deficits and depressed states in patients with schizophrenia.

The study uses a digital phenotype platform called *Health Outcomes Through Positive Engagement and Self-Empowerment* (HOPES) that is based on the open-source Beiwe platform but includes smartphone data in addition to wearable devices. Previous research on the Beiwe platform showed that its detection rate for behavioral anomalies in the 2 weeks preceding a relapse was 71% higher than that in other time periods [26,27]. The first phase of HOPES entails observing participant behavior with the primary goal of developing machine learning algorithms that can predict relapse or readmission within 6 months. The second phase will include sending timely interventions in response to recognized pattern signatures, such as early warnings of relapse that may give participants the time to take steps toward preventing relapse [24,28]. Preliminary data from the wrist wearables of 21 patients over 1 week have been published and align with past correlations with PANSS scores, though these data are limited by the short time frame [29]. Once completed, the study will likely build upon its predecessor’s ability to detect relapse while testing the efficacy of wearable-based interventions based on real-time data.

Other platforms are being studied as well, such as Mobile Therapeutic Attention for Treatment-Resistant Schizophrenia (m-RESIST)—a project that is currently testing the feasibility of its platform, wearable, and smartphone app combination [12,30]. Likewise, the Sleepsight platform was used with wearable sleep and activity data from 15 people with schizophrenia living in their homes. The protocol was demonstrated to be a feasible operation that was acceptable by patients [8].

Wearables offer the same mainstream health and fitness benefits to patients with schizophrenia. Bueno-Antequera et al [31] gave 82 outpatients with schizophrenia an armband sensor for 1 week to measure sedentary behavior and estimate cardiorespiratory fitness based on a 6-minute walking test. The results showed consistent relationships among sedentary behavior, increased BMI, and reduced cardiorespiratory fitness. These relationships remained significant even when controlling for age, symptom severity, and antipsychotic medication. Further, a clinical trial is currently testing wearables as part of 2 lifestyle interventions for young adults who are considered overweight or obese based on their BMIs and have a diagnosis of schizophrenia, schizoaffective disorder, bipolar disorder, or major depressive disorder [32]. The trial is expected to be completed by May 2022 and is likely to shed more light on the potential of wearables to address the lifestyle-related health issues of patients with psychiatric illness [33].

## Future Directions

Wearables and machine learning have already made extensive progress, as the previously mentioned studies indicate, but several changes have been proposed to streamline the path for future research. For instance, the World Health Organization has published the Mobile Health Evaluation, Reporting and Assessment (mERA) checklist, which highlights 16 criteria to guide experimental design and study interpretation, including user feedback, accessibility, replicability (requiring open-source code), scalability, cost assessment, and data security [34]. A surprising number of studies do not meet these criteria, and only 4 of 11 studies on mobile health and smartphone apps for schizophrenia reported on data security in a 2017 review [35]. The universal adoption of these guidelines may also make it possible for a database to be generated that would allow researchers to input and sort all contributed data by device type or patient population. By pooling data, the rate of reliability issues can be reduced and statistical power can be increased to overcome the common issue of having a small sample size.

Numerous studies have evaluated the validity and feasibility of wearables in psychiatric populations, but 2 adaptations may be beneficial for future research. First, there is variation among studies in what technology an investigated wearable is validated against, whether it be an actigraphy watch or the gold standard—polysomnography [9,10]. Although initial validations against other devices are more accessible for researchers and convenient for patients, especially in early research, studies have overall confirmed the validity of such wearables in these contexts. Thus, future validity studies should aim for comparisons between wearable technology and gold standards when possible.

As mentioned before, there is an abundance of validity, feasibility, and acceptability studies in the literature, as is characteristic of early research. Multiple consumer and clinical-grade devices have been successfully validated against each other as well as the gold standard, and studies can now shift toward an increasingly clinical emphasis. A 2019 review of smartphone sleep tracking in psychiatric populations showed that very few studies reported on improvements in sleep or mental health outcomes. Aledavood et al [36] recommended a patient-centered approach that prioritizes metrics, such as improving adherence, predicting risk and relapse, and determining whether these advances result in meaningful benefits for patients [36]. This approach shares similarities with the mERA checklist.

Lastly, there are tablet-based tests that are already in clinical use, such as the Cambridge Neuropsychological Test for identifying cognitive deficits and the Brief Assessment of Cognition in Schizophrenia, which can differentiate patients with severe mental illness from controls. The CogniSense app (Quest Diagnostics Incorporates) is likewise designed for tablets and has shown diagnostic rates that are equal to or higher than those of the Mini-Mental State Exam and the Mini-Cog Exam for cognitive impairment. Researchers, clinicians, and patients would all benefit if these exams were tailored to the small screen of the typical wearable [13,37]. Even if it were necessary to use condensed versions of these assessments, a wearable may still provide insights and improve patient participation through accessible reminders and the convenient completion of assessments from one’s wrist. The results could then be sent to clinicians or researchers as a readout of all metrics at the time of the assessment.

## Conclusions

Wearables in schizophrenia have made incredible progress in just the past 3 years. The foundation for wearables was further established by studies supporting the validity of various devices in their ability to track sleep, which is especially useful in schizophrenia, as sleep disturbances may be predictive of disease onset or the acute worsening of psychotic symptoms. Through machine learning, an analysis of heart rate and motor activity can be conducted to differentiate between controls and patients with schizophrenia. These and other metrics capture the autonomic dysregulation detected when patients are actively experiencing paranoia, hallucinations, or delusions. Several platforms that are currently being tested, such as HOPES, m-RESIST, and Sleepsight, may ultimately link wearable-derived patient data to clinicians. The future is bright for wearables in schizophrenia, as the recent literature exemplifies their potential to offer real-time insights to guide diagnosis and management.

## Abbreviations

FEP: first-episode psychosis
HOPES: Health Outcomes Through Positive Engagement and Self-Empowerment
m-RESIST: Mobile Therapeutic Attention for Treatment-Resistant Schizophrenia
mERA: Mobile Health Evaluation, Reporting and Assessment
PANSS: Positive and Negative Syndrome Scale
SAM: social ambience measure
